# Bystander Effects and Profibrotic Interactions in Hepatic Stellate Cells during HIV and HCV Coinfection

**DOI:** 10.1155/2024/6343757

**Published:** 2024-04-30

**Authors:** Cintia Cevallos, Patricio Jarmoluk, Franco Sviercz, Cinthya A. M. López, Rosa N. Freiberger, M. Victoria Delpino, Jorge Quarleri

**Affiliations:** Consejo de Investigaciones Científicas y Técnicas (CONICET), Instituto de Investigaciones Biomédicas en Retrovirus y Sida (INBIRS), Universidad de Buenos Aires (UBA), Paraguay, 2155, piso 11, C1121 ABG, Buenos Aires, Argentina

## Abstract

This study aims to explore the influence of coinfection with HCV and HIV on hepatic fibrosis. A coculture system was set up to actively replicate both viruses, incorporating CD4 T lymphocytes (Jurkat), hepatic stellate cells (LX-2), and hepatocytes (Huh7.5). LX-2 cells' susceptibility to HIV infection was assessed through measurements of HIV receptor expression, exposure to cell-free virus, and cell-to-cell contact with HIV-infected Jurkat cells. The study evaluated profibrotic parameters, including programed cell death, ROS imbalance, cytokines (IL-6, TGF-*β*, and TNF-*α*), and extracellular matrix components (collagen, *α*-SMA, and MMP-9). The impact of HCV infection on LX-2/HIV-Jurkat was examined using soluble factors released from HCV-infected hepatocytes. Despite LX-2 cells being nonsusceptible to direct HIV infection, bystander effects were observed, leading to increased oxidative stress and dysregulated profibrotic cytokine release. Coculture with HIV-infected Jurkat cells intensified hepatic fibrosis, redox imbalance, expression of profibrotic cytokines, and extracellular matrix production. Conversely, HCV-infected Huh7.5 cells exhibited elevated profibrotic gene transcriptions but without measurable effects on the LX-2/HIV-Jurkat coculture. This study highlights how HIV-infected lymphocytes worsen hepatic fibrosis during HCV/HIV coinfection. They increase oxidative stress, profibrotic cytokine levels, and extracellular matrix production in hepatic stellate cells through direct contact and soluble factors. These insights offer valuable potential therapies for coinfected individuals.

## 1. Introduction

Due to a common transmission route involving infected human blood, coinfections of the HCV and human immunodeficiency virus (HIV) are relatively prevalent, with an estimated 2.3 million individuals globally living with HCV/HIV coinfection [1]. Numerous studies have evidenced that HIV infection expedites the progression of HCV infection-induced hepatic fibrosis [2, 3]. The underlying pathogenesis of accelerated hepatic fibrosis in individuals coinfected with HIV and HCV remains unclear but is likely intricate, potentially involving multiple factors such as direct viral effects, immune/cytokine dysregulation, and heightened oxidative stress [4].

Chronic liver injury triggers hepatic fibrosis, marked by abnormal synthesis and accumulation of extracellular matrix (ECM) proteins [5]. Key contributors to fibrogenesis include HSCs, hepatocytes, and various nonparenchymal cells, including immune cells. Even in homeostasis, immune cells are dispersed in liver tissue. Following injury, compromised cells release inflammatory mediators, recruiting leukocytes to the injury site [6]. Simultaneously, lymphocytes release cytokines, activating macrophages and fibroblasts, perpetuating inflammation [7]. Sustained injury signals prompt quiescent HSC to swiftly activate, becoming myofibroblast-like [8, 9]. Activated cells migrate to repair sites, releasing profibrogenic cytokines and transforming into myofibroblasts expressing alpha-smooth muscle actin (*α*-SMA) and secreting ECM proteins. This imbalance fuels liver fibrogenesis, potentially leading to cirrhosis and HCC [7, 8].

The objective of this study was to elucidate the impact of HIV infection and HCV/HIV coinfection on the acceleration of hepatic fibrosis by activating HSC, involving intercellular communication among different hepatic cell types. For this goal, a cell culture system that facilitates HSC-T lymphocyte contact (permissive for HIV infection) accompanied by a conditioned medium derived from HCV-infected hepatocytes was used. The findings indicate that active HIV replication in T lymphocytes plays a role in the fibrogenic response from HSC, a process further intensified by HCV replication in hepatocytes.

## 2. Methods

### 2.1. Experimental Timeline Schedule

**Figure 1:**
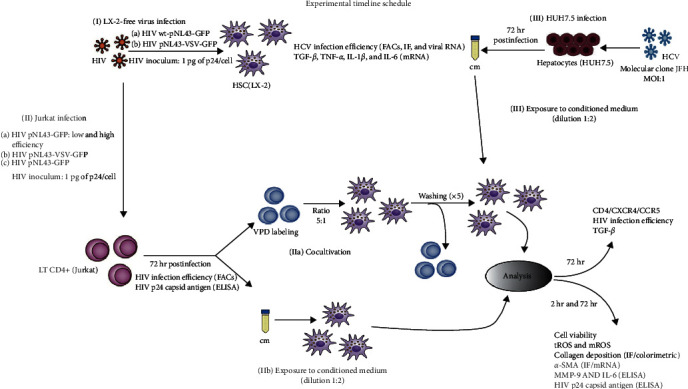
Experimental timeline schedule. The permissiveness of HSCs (LX-2) to HIV infection was assessed using two strategies: (I) cell-free virus (pNL43-GFP) and (IIa) HIV-infected Jurkat cells with pNL43-GFP in coculture with LX-2 (ratio 5:1). LX-2 cells were exposed to HIV during 5 hr and washed. After 72 hr in culture, the kinetics of HIV replication was evaluated on LX-2 cells by quantifying the HIV-p24 antigen in supernatants utilizing an ELISA kit, and the infection efficiency by quantifying GFP+ cells by flow cytometry was evaluated. (IIb) Moreover, the effect of supernatant collected at 72 hr from HIV-infected LT CD4+ cells (Jurkat)—“cm Jurkat (HIV)”—on LX-2 was evaluated. Complementary, (III) the effect of HCV was assessed by exposing the LX-2/LT HIV cocultures to a conditioned medium (supernatant collected at 72 hr from HCV-infected hepatocytes (Huh7.5)—“cm Huh/HCV”—immediately after cocultivation. The different determinations on LX-2 cells were performed at 2 or 72 hr post-coculture or exposure to conditioned medium. The percentage of infection on Jurkat and Huh7.5 cells was evaluated at 72 hr postinfection and immediately before cocultivation or conditioned medium stimulation. LX-2 and Jurkat cells were infected to cell-free virus (1 pg/cell) and Huh7.5 cells were infected with a MOI: 1.

The experimental design and timeline schedule are summarized in Figure 1.

### 2.2. Cell Culture

The LX-2 human hepatic stellate cell line, graciously provided by Dr. Scott L. Friedman (Mount Sinai School of Medicine, New York, NY, USA) [10], was cultured in Dulbecco's Modified Eagle Medium (DMEM) supplemented with 2% fetal bovine serum (FBS), L-glutamine (2 mM), 100 U/mL penicillin, and 100 *µ*g/mL streptomycin (all from Life Technologies) at 37°C and with 5% CO_2_. For investigating HSC transdifferentiation, LX-2 cells were cultured in DMEM supplemented with 2% FBS (quiescent culture) or 10% FBS (activated culture), the latter serving as a positive control.

The human CD4+ T lymphocyte line Jurkat, obtained from the American Type Culture Collection (ATCC, USA), was cultured in RPMI 1640 medium supplemented with 10% FBS, L-glutamine (2 mM), 100 U/mL penicillin, and 100 *µ*g/mL streptomycin at 37°C and with 5% CO_2_.

The human Huh7.5 hepatocellular carcinoma-derived cell line was obtained from ATCC. It was cultured in DMEM supplemented with 10% FBS and L-glutamine (2 mM), 100 U/mL penicillin at 37°C and with 5% CO_2_, and 100 *µ*g/mL streptomycin. After Jurkat-LX-2 cocultivation, the stimulation of LX-2 cells with conditioned medium from Huh7.5 cells was performed at a half dilution.

### 2.3. Viral Infections

As depicted in Figure 1, the LX-2 and Jurkat cell lines were exposed to wild-type HIV X4-tropic NL4.3-eGFP molecular clone, carrying the enhanced green fluorescent protein gene (eGFP) and an internal ribosome entry site (IRES) upstream of Nef reading frames [11]. Alternatively, cell-free pseudotyped HIV, coexpressing G glycoprotein from vesicular stomatitis virus (VSV-G), was also used [11]. Additionally, Jurkat cells were exposed to pNL4.3 wild-type HIV molecular clone.

LX-2 cells were seeded at 50,000 cells/well in 24-well plates and exposed to an inoculum of 1 pg of p24/cell either wild-type NL4.3-eGFP or pseudotyped HIV. After 4 hr of virus exposure at 37°C, the cells were washed five times with phosphate-buffered saline (PBS) and incubated in a fresh culture medium at 37°C and with 5% CO_2_ for 72 hr.

For Jurkat infection, cells were seeded in a 96-well culture plate at 100,000 cells/well and infected with 1 pg of p24/cell of HIV-X4-wild-type or HIV-X4-GFP. HIV infection was carried out using two approaches: (i) simple virus–cell mixing at 37°C for 18 hr, followed by washing three times with PBS and incubation in fresh culture medium at 37°C, and (ii) spinoculation to emulate efficient cell-to-cell virus spread [12]. Pseudotyped HIV-VSV-G stock was used as a control. After 72 hr, the HIV infection was monitored in three different ways: (1) HIV capsid protein p24 release in cell culture supernatants by ELISA assay (INNOTEST® HIV Antigen mAb), (2) intracellular expression of p24 (PE-KC57, #cat 6604667 or FITC-KC57, #cat 6604665, Beckman Coulter), and (3) HIV-GFP gene expression by flow cytometry. Jurkat cell culture supernatants collected at 72 hr postinfection (hpi) were stored at −80°C for use as a conditioned medium (cm).

When HIV-infected Jurkat cells were intended to be used in cell-to-cell contact, these cells were washed, counted, and labeled with Violet Proliferation Dye 450 (VPD 450, BD) and then cocultured with LX-2 for 5 hr without FBS added. Then, the cell coculture was washed five times with PBS to minimize residual Jurkat cells. Subsequently, LX-2 cells were incubated in a fresh culture medium with 2% FBS at 37°C for 72 hr.

To determine LX-2 susceptibility to HIV infection, two variables were monitored 72 hr post-cocultivation: (1) HIV capsid protein p24 in cell culture supernatants and (2) HIV gene expression by flow cytometry GFP measurement. Control experiments included LX-2 cultures and cocultures with uninfected HIV Jurkat labeled with VPD.

An HCV virus stock was prepared from the J6/JFH clone obtained from Apath LLC (USA), and its infectivity was assessed using a fluorescence method in Huh7.5 cells [13, 14]. Serial dilutions of the HCV viral stock were incubated with Huh7.5 cells for 4 days, fixed, and immunostained with HCV core antibody (1:1000 #cat. ab58713, Abcam). The infectious HCV titer was determined [15], and viral RNA in the HCV stocks was quantified using a commercial assay (COBAS® AmpliPrep/COBAS® TaqMan® HCV Test, v2.0). For all experiments, Huh7.5 cells were infected with HCV at a multiplicity of infection (MOI) = 1. All experiments adhered to BSL-3 laboratory standards at INBIRS, with biological materials autoclaved and incinerated following institutional rules.

### 2.4. Cellular Parameters Measured by Flow Cytometry

- Cell death: Programed cell death (PCD) levels were evaluated by measuring the sum of apoptotic and necrotic cells using dual staining with APC-conjugated annexin-V and 7-AAD using the Annexin V/7-AAD Apoptosis Detection Kit (BD Biosciences). Staurosporine (STS) at 1 *μ*M concentration was used as cell death positive control.
- CD4, CCR5, and CXCR4 expression on LX-2 cells: It was analyzed through staining with specific antibodies APC-labeled anti-human CCR5 (1:5 #cat. ab176536, Abcam), PE-labeled anti-human CXCR4 antibody (1:5 #cat. 555974 BD Pharmingen), and PerCP-labeled anti-human CD4 (1:100 #cat. 344624 BioLegend) and flow cytometry.
- Reactive oxygen species (ROS) production:
- Total cellular ROS (tROS) production: Assessed using the 2′,7′-dichlorodihydrofluorescein diacetate (H2DCFDA, Abcam) assay for total cellular ROS production, including hydroxyl, peroxyl, and other ROS. Cells were incubated with DCFDA at 10 *µ*M in essential medium at 37°C for 45 min and then evaluated by flow cytometry. Tert-butyl hydroperoxide solution (TBH) at 100 *μ*M concentration was used for tROS production as positive control.
- Mitochondrial ROS (mROS) generation: Quantified by flow cytometry in cells stained by 5 *µ*M MitoSOX™ (ThermoFisher Scientific) for 15 min. Rotenone at 10 *μ*M concentration was used for mROS production as positive control.
- TGF-*β* producing cells: LX-2 cells were fixed, permeabilized, and stained to detect TGF-*β*1 production (1:10, #cat. 562339 BD Pharmingen).
- Flow cytometry measurements were carried out using a FACSCanto (BD Biosciences), and data analysis was conducted using FlowJo X software (TreeStar).

### 2.5. Assessment of Collagen Deposition via Sirius Red Staining

Collagen deposition in LX-2 cells was assessed using Sirius Red (Sigma–Aldrich). After fixing the LX-2 layers with Bouin's fluid, the plates underwent washes and air-drying before applying the Sirius Red dye reagent. Staining occurred for 18 hr, followed by thorough washing to eliminate unbound dye with 0.01 N hydrochloric acid. For quantitative analysis, the stained material was dissolved in 0.1 N sodium hydroxide, and the optical density (OD) was measured at 550 nm using a microplate reader (Metertech, Inc.). The measurements were recorded against 0.1 N sodium hydroxide as a blank.

### 2.6. Determination of *α*-SMA and Collagen Production Using Immunofluorescence Microscopy

Infected LX-2 cells were fixed at 72 hpi in 4% paraformaldehyde for 15 min at room temperature and permeabilized with 0.3% Triton X-100 (Roche Diagnostics) for 10 min. Cells were then incubated overnight at 4°C with anti-*α*-SMA (1:100 #cat. PA5-19465, Thermo Fisher Scientific) diluted in PBS Tween (0.025%) and 1% BSA. Then cells were washed with PBS and incubated with Alexa Fluor 647-conjugated goat-anti-rabbit IgG (H + L) secondary antibody (1:100 #cat. ab150115 Abcam, UK) in PBS at room temperature in the dark. For collagen I deposition in the infected fixed cells, incubation with anti-collagen I labeled with Alexa Fluor 488 (1:100 #cat. ab275996 Abcam, UK) in PBS Tween (0.025%) and 1% BSA also occurred overnight at 4°C. Nuclear counterstaining was achieved using 4,6-diamidino-2-phenylindole (DAPI) (Molecular Probes). After PBS washing, coverslips were mounted in PBS–glycerin (9:1 v/v) and analyzed using a Zeiss LSM 800 confocal microscope (Zeiss, Germany).

### 2.7. Cellular RNA Extraction and Quantitative Real-Time PCR

Total RNA was extracted from LX-2 and Jurkat cells using the kit Quick-RNA MiniPrep Kit (Zymo Research) according to the manufacturer's instructions. Washed LX-2 cells were harvested by a scraper for RNA extraction. cDNA was synthesized from 1 *µ*g of total RNA using the reverse transcriptase Improm-II enzyme (Promega). Real-time PCR was performed using SYBR green as a DNA-binding fluorescent dye and a StepOne Real-Time PCR System (Applied Biosystems). The following primers pair were used: *α*-SMA: sense 5-CGTGGCTATTCCTTCGTTAC-3′, antisense 5′-TGCCAGCAGACTCCATCC-3′; TGF-*β*: sense 5′-GGACACCAACTATTGCTTAG-3′, antisense 5′-TCCAGGCTCCAAATGTAGG-3′; TNF-*α*: sense 5′-CCGAGGCAGTCAGATCATCTT-3′, antisense 5′-AGCTGCCCCTCAGCTTGA-3′; IL-6: sense 5′-AGACAGCCACTCACCTCTTCAG-3′, antisense 5′-TTCTGCCAGTGCCTCTTTGCTG-3′; IL−1*β*: sense 5′- CCACAGACCTTCCAGGAGAATG-3′, antisense 5′-GTGCAGTTCAGTGATCGTACAGG-3′; and GAPDH: sense 5′-GTCAGTGGTGGACCTGACCT-3′, antisense 5′-TGCTGTAGCCAAATTCGTTG-3′.

The amplification cycles were 95°C for 15 s, 55°C for 30 s, and 72°C for 60 s.

Melting curve analysis was then performed. All primer sets yielded a single product of the correct size. The fold change (relative expression) in gene expression was calculated using the relative quantification method (2^−*ΔΔ*Ct^) [16]. Relative expression levels were normalized against GAPDH. Intraexperiment Ct value differences between samples were less than 0.5.

### 2.8. Measurement of IL-6 and MMP-9 Concentration

Human IL-6 was determined through a sandwich enzyme-linked immunosorbent assay using paired cytokine-specific monoclonal antibodies. The assay was performed according to the manufacturer's instructions (BD Pharmingen). MMP-9 levels were quantified using an ELISA kit (Quantikine, R&D, Catalog #: DMP900), following the manufacturer's instructions.

### 2.9. Statistical Analysis

Statistical analysis was performed wherever applicable. Statistical analysis was performed with one-way ANOVA. Multiple comparisons between all pairs of groups were made using Tukey's test, and those between two groups were made using the Mann–Whitney *U* test. Graphical and statistical analyses were performed with GraphPad Prism 8.0 software. Each experiment was performed in triplicate (technical replicates) with different culture preparations on two to four independent occasions (biological replicates). Data were represented as mean ± SD measured in triplicate from three individual experiments. A *p*  < 0.05 is represented as  ^*∗*^, *p*  < 0.01 as  ^*∗∗*^, *p*  < 0.001 as  ^*∗∗∗*^, and *p*  < 0.0001 as  ^*∗∗∗∗*^. A statistically significant difference between groups was accepted at a minimum level of *p*  < 0.05.

## 3. Results

### 3.1. LX-2 Cells are Nonpermissive to HIV Wild-Type Infection through Either Exposure to Cell-Free Virus or Transmission via Cell-to-Cell Contact from HIV-Infected Jurkat Cells

**Figure 2:**
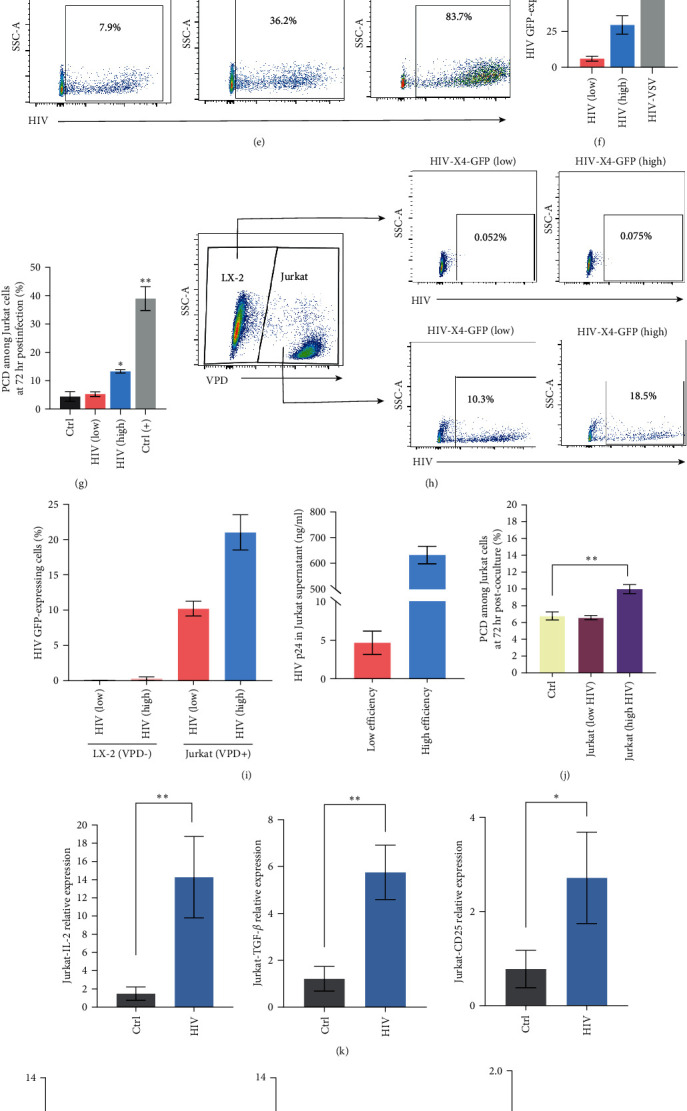
Evaluation of the susceptibility and permissiveness of LX-2 cells to HIV infection by flow cytometry. (a) Representative flow cytometry dot plots showing the relative percentages of LX-2 infection after 72 hr. On the left, LX-2 cells exposed to HIV-X4-GFP showed undetectable GFP expression depicting them as not susceptible or permissive to HIV infection after cell-free virus challenge. On the right, the dot plot shows that LX-2 cells are permissive to pseudotyped HIV replication, after exposure to free HIV-VSV-GFP. (b) Bar graph representing the percentage of infection obtained (0.0744 ± 0.05) and (10.85% ± 1.14%), respectively. (c) Viral infection was evaluated by measuring p24 antigen in culture supernatants from LX-2 cells exposed to free HIV-X4 and HIV-VSV. After 72 hr (1.63 ± 011 ng/mL and 21.67 ± 3.61, respectively). (d) Bar graph exhibiting poor coexpression of CD4 receptor, CXCR4, and CCR5 coreceptors measured on LX-2: 0.47% ± 0.13%, and CD4/CXCR4: 0.33% ± 0.15%, respectively. These determinations were performed by flow cytometry. (e) Representative flow cytometry dot plots showing the efficiency of Jurkat cell infection at 72 hr according to the method used: HIV-X4-GFP-free virus infection (low), HIV-X4-GFP spinoculation infection (high), and HIV-VSV-GFP-free virus infection. (f) Bar graphs depicting the corresponding infection percentages for “low” (6.1% ± 1.7%), “high” (29.5% ± 6.4%), and HIV-VSV-GFP (85.1% ± 7.1%) respectively. (g) Programed cell death levels (PCD) (positive staining for annexin V and/or 7-AAD) in Jurkat control nonexposed (Ctrl), Jurkat (HIV) (low), and Jurkat HIV (high). The PCD was significantly higher in Jurkat (HIV) (high) relating to Jurkat (HIV) (low) *p*=0.02. (h) Cocultivation between VPD (−) LX-2 as target cells and VPD (+)-labeled HIV-exposed Jurkat (inoculum: 1 pg of p24/cell) as donor cells. Representative flow cytometry dot plots showing the LX-2 VPD (−) and Jurkat VPD (+) after 72 hr post-cocultivation. In addition, the dot plots depicting the HIV infection level for both populations reveal that LX-2 cells remain uninfected at 72 hr post-cocultivation, regardless of the infection efficiency of the cocultivated Jurkat cells. (i) Percentage of cells expressing HIV—GFP for LX-2 VPD (−) HIV low: 0.047% ± 0.015%; LX-2 VPD (−) HIV high: 0.238% ± 0.31%; Jurkat VPD (+) HIV low: 10.2% ± 1%; and Jurkat VPD (+) HIV high: 21% ± 2.5% and HIV replication in Jurkat cells (measured as p24 antigen in supernatants). Low-grade: 4.68 ± 1.51 ng/mL; high-grade: 632.83 ± 34 ng/mL. (j) Programed cell death levels (PCD) (positive staining for annexin V and/or 7-AAD) in Jurkat control nonexposed (Ctrl), Jurkat (HIV) (low), and Jurkat HIV (high) after 72 hr of coculture. The PCD was significantly higher in Jurkat (HIV) (high) relating to noninfected Jurkat (Ctrl) *p*=0.002. (k) Bar graph showing an increase in the IL-2, CD25, and TGF-*β* expression in HIV-infected Jurkat cells vs. uninfected Jurkat (14.27 ± 4.47, 2.71 ± 0.96, and 5.76 ± 1.16, respectively), measured by RT-qPCR at 72 hr postinfection. (l) Bar graphs showing flow cytometry measurement of the CD4, CXCR4, and CXCR5 percentage expression on LX-2 at 72 hr post-cocultivation or conditioned medium exposition. CXCR4 levels in Jurkat (HIV): LX-2 cocultivation vs. Jurkat (NI): LX-2 cocultivation *p*=0.0164; CCR5 levels in Jurkat (HIV): LX-2 cocultivation vs. Jurkat (NI): LX-2 cocultivation *p*  < 0.0001; CD4 levels remained unchanged for any treatment. NS, not significant. Graphics are showing values obtained from three independent experiments. Data are given as the mean ± SD,  ^*∗*^*p*  < 0.05,  ^*∗∗*^*p*  < 0.01,  ^*∗∗∗*^*p*  < 0.001, and  ^*∗∗∗∗*^*p*  < 0.0001.

The susceptibility and permissiveness of LX-2 cells to HIV infection were evaluated through different approaches. Initially, when exposed to cell-free virus (1 pg of p24/cell), LX-2 cells did not exhibit infection (Figure 2(a)–2(c)). To further understand their susceptibility, the surface expression of HIV receptor/coreceptor molecules (CD4, CXCR4, and CCR5) was measured by flow cytometry, revealing a low coexpression level of these receptors among LX-2 cells, such as CD4/CCR5: 0.47% ± 0.13%, and CD4/CXCR4: 0.33% ± 0.15% (Figure 2(d)). In this context, the permissiveness of LX-2 cells to HIV replication was assessed using cell-free pseudotyped HIV-VSV-G that entered the cell through a clathrin-mediated endocytic pathway [17], showing an efficiency of 10.85% ± 1.14% (Figure 2(a)–2(c)).

Previous studies performed in vitro explored the transfer of HIV between T cells demonstrating high efficiency, capable of surpassing cell-free infection [18]. Moreover, it is known that HSCs may contribute to the migration of CD4+ T cells into the liver parenchyma [19]. LX-2 cells were cultured with HIV-infected Jurkat cells. The HIV Jurkat infection performed through different infection strategies resulted in two distinct infection efficiencies named “low” and “high” (6.1% ± 1.7% and 29.5% ± 6.4%, respectively). Furthermore, when Jurkat cells were infected with pseudotyped HIV-VSV-G, a remarkably high efficiency was achieved, reaching 85.1% ± 7.1% (Figures 2(e) and 2(f)). Notably, as depicted in Figure 2(g), Jurkat cell programed cell death (PCD) rates at 72 hpi correlated with infection efficiency, with low and high infection levels showing PCD levels of 5.26% ± 0.84% and 13.30% ± 0.58%, respectively, PCD level among noninfected Jurkat cells (control) was 4.39% ± 1.22%.

Flow cytometry analysis of cocultures, with Jurkat cells labeled with VPD for distinction, demonstrated that LX-2 cells remained uninfected at 72 hr post-coculture, regardless of the infection efficiency of the cocultured Jurkat cells (Figures 2(h) and 2(i)). At this time point, Jurkat cells' PCD level was 6.78% ± 0.49%, 6.59% ± 0.24%, and 9.99% ± 0.53% for noninfected (control), low-efficiency, and high-efficiency HIV-infected Jurkat cells, respectively (Figure 2(j)).

HIV-mediated activation of Jurkat cells was evidenced by increased mRNA levels of IL-2, CD25, and TGF-*β* (Figure 2(k)), for those with higher infection levels; therefore, the subsequent experiments were conducted to study the impact of this condition on LX-2 cells.

Notably, the expression of CXCR4 and CCR5 in LX-2 cells increased after 3 days of contact with HIV-infected Jurkat cells, but not with “cm,” while CD4 expression remained unchanged (Figure 2(l)).

In summary, LX-2 cells exhibit resistance to HIV infection, whether exposed to free virus or in contact with activated and infected lymphocytes, even with high efficiency. This resistance is supported by the low CD4/CXCR4/CCR5 receptor expression. However, through a CD4/CXCR4/CCR5-independent virus entering route, LX-2 cells display permissiveness to HIV replication.

### 3.2. Paradoxically, HIV-Infected CD4 T Lymphocytes Can Induce Oxidative Stress in LX-2 without Altering Cell Viability

Endogenous reactive oxygen species (ROS), as well as those generated by infiltrating inflammatory cells, trigger the activation of HSCs [20, 21].

**Figure 3:**
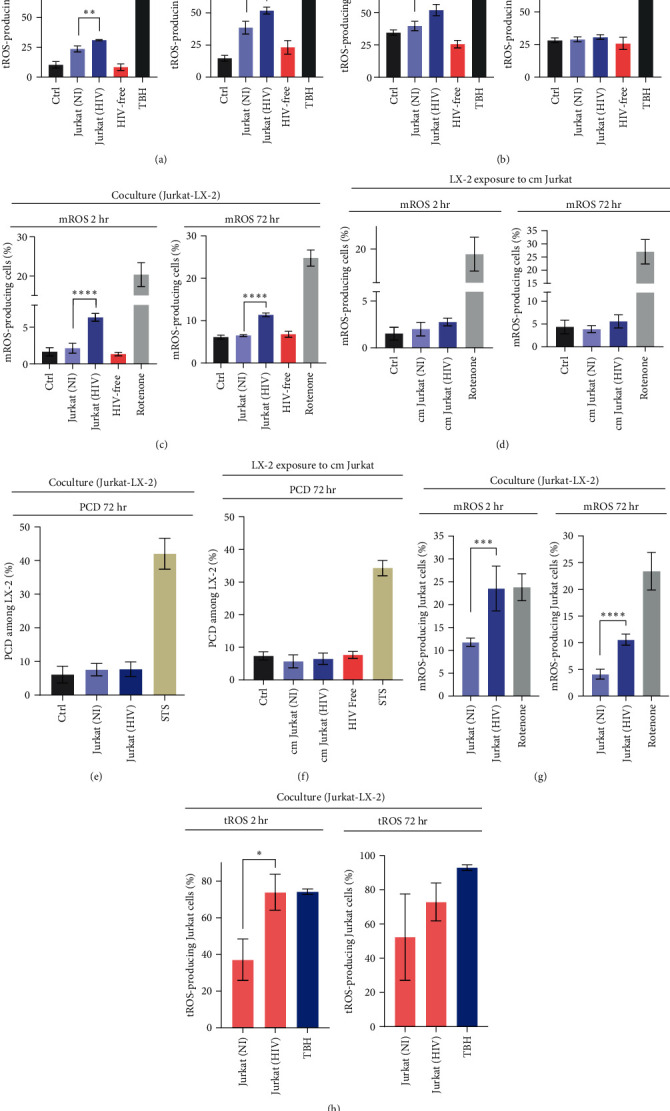
Analysis of ROS production among LX-2 after 2 and 72 hr post-cocultivation or conditioned medium exposition by flow cytometry. (a) tROS generation by LX-2 cells after cocultivation with Jurkat (HIV) was increased to the control (LX-2 cocultivated with uninfected Jurkat) for both 2 and 72 hr, (*p*  < 0.01 and *p*  < 0.001, respectively). (b) tROS generation by LX-2 cells after “cm” from Jurkat (HIV) was higher in comparison with control (LX-2 exposure “cm” from uninfected Jurkat) at 2 hr, *p*  < 0.001. At 72 hr, no differences were observed. (c) mROS production by LX-2 cells after cocultivation with Jurkat (HIV) was greater than control (LX-2 cocultivated with uninfected Jurkat) for both 2 and 72 hr, *p*  < 0.0001. (d) mROS generation by LX-2 cells after conditioned “cm” from Jurkat (HIV) remained unchanged for the different conditions evaluated for both 2 and 72 hr.(e, f) Programed cell death levels (PCD) (positive staining for annexin V and/or 7-AAD) among LX-2 after 72 hr of cocultivation and “cm” exposition remained unchanged for the different conditions evaluated. (g) mROS production by HIV-infected Jurkat cells after cocultivation with LX-2 was greater than control (uninfected Jurkat cocultivated with LX-2) for both 2 hr (*p*  < 0.001) and 72 hr (*p*  < 0.0001). (h) tROS generation by HIV-infected Jurkat cells after cocultivation with LX-2 was increased to the control (uninfected Jurkat cocultivated with LX-2) for 2 hr (*p*  < 0.05) but not at 72 hr. cm, conditioned medium; NS, not significant; STS, staurosporine; and TBH, tert-butyl hydroperoxide. Graphics are showing values obtained from three independent experiments. Data are given as the mean ± SD.  ^*∗*^*p*  < 0.05,  ^*∗∗*^*p*  < 0.01,  ^*∗∗∗*^*p*  < 0.001, and  ^*∗∗∗∗*^*p*  < 0.0001.

The correlation between HIV exposure (via cell-free virus or cell-to-cell contact) and the generation of reactive oxygen species (ROS) in LX-2 cells was studied. Using a redox-sensitive fluorescent probe (DCFDA), total ROS (tROS) levels were assessed at 2 and 72 hr post-HIV exposure (hpi) via flow cytometry. At 2 hpi, LX-2 cells exhibited a threefold increase in tROS production when cocultured with HIV-infected Jurkat cells, reaching 31.0% ± 0.5%, but not with cell-free HIV. Subsequently, tROS levels remained significantly elevated threefold at 72 hpi (Figure 3(a)). Similarly, exposure of LX-2 cells to conditioned media from Jurkat cells led to a significant but lower increase in tROS levels, observed only at 2 hpi (1.5-fold) (Figure 3(b)).

Mitochondria, as the primary source of reactive oxygen species (mROS) in cells, were examined using a specific fluorogenic dye, MitoSOX. In Figure 3(c), LX-2 cells in contact with HIV-infected Jurkat cells showed significantly higher mROS levels at 2 hpi and 72 hpi (3.9-fold and 2.3-fold, respectively). Exposure to “cm Jurkat (HIV)” did not produce changes in the mROS generation, either at 2 or 72 hr (Figure 3(d)). Notably, HIV-free particles did not disturb mROS balance in LX-2 cells. Elevated cellular ROS levels can induce damage [22], yet LX-2 cell viability remained unchanged when cocultured with HIV-infected Jurkat cells or exposed to their “cm” (Figures 3(e) and 3(f)). In the coculture, Jurkat cells exhibited significantly higher ROS levels (total and mitochondrial) when they were infected with high efficiency (Figures 3(g) and 3(h)).

These results suggest increased oxidative stress in LX-2 cells exposed to soluble factors from HIV-infected Jurkat cells and more pronounced through cell-to-cell contact. Paradoxically, this does not align with a simultaneous rise in programed cell death levels in HSCs.

### 3.3. HIV-Infected CD4 T Lymphocytes Can Modulate the Production of Extracellular Matrix Components (Collagen, *α*-SMA, and MMP-9) and the Release of Profibrotic Factors by LX-2

**Figure 4:**
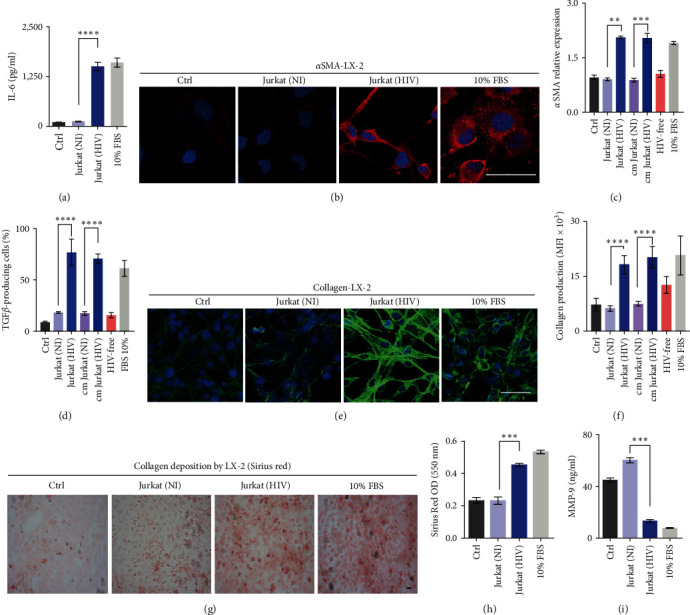
Determination of profibrotic and proinflammatory parameters. (a) Determination of IL-6 secretion by LX-2 cells implementing ELISA kit. Seventy-two hours post-cocultivation with Jurkat (HIV), the IL-6 production was higher than control (LX-2 cocultivated with uninfected Jurkat) *p*  < 0.0001. (b, c) Determination of *α*-SMA production by LX-2 revealed by immunofluorescence with a specific antibody and real-time PCR. The *α*-SMA production (protein expression and mRNA levels) was increased 1.2-fold when LX-2 cells were cocultured with Jurkat (HIV) exposure to “cm” from Jurkat (HIV) in comparison with the corresponding controls (LX-2 cocultivated with uninfected Jurkat and LX-2 exposure “cm” from uninfected Jurkat). (d) In the same way, the intracellular production of TGF-*β* by LX-2 cells (determined by flow cytometry) was increased up to 4.3-fold for both conditions from HIV-infected Jurkat cells (“cm” and cell-to-cell contact). (e–h) Determination of collagen deposition by LX-2 cocultured with Jurkat (HIV) or exposure to conditioned medium “cm” from Jurkat (HIV) was a threefold increase compared to corresponding controls. Such determination was performed utilizing immunofluorescence microscopy and Sirius Red staining. (i) MMP-9 levels quantified in LX-2 cocultured with Jurkat (HIV) or exposure to “cm” from Jurkat (HIV) culture supernatant implementing an ELISA kit were downregulated (4.3-fold). NI, noninfected; cm, conditioned medium); and NS, not significant). Scale bar: 50 *µ*m. Graphics are showing values obtained from three independent experiments. Data are given as the mean ± SD.  ^*∗∗*^*p*  < 0.01,  ^*∗∗∗*^*p*  < 0.001, and  ^*∗∗∗∗*^*p*  < 0.0001.

HSCs are pivotal in liver fibrosis, undergoing transformation into myofibroblasts and regulated by key mediators like IL-6 and TGF-*β*. In this study, LX-2 cells were exposed to conditioned media or cocultured with high-efficiency HIV-infected Jurkat cells for 72 hr. The results obtained indicate that under these conditions, IL-6 secretion by LX-2 was induced at similar levels to those observed with its culture in 10% FBS, which served as a positive control (12.5-fold increase) (Figure 4(a)). Subsequently, LX-2 transdifferentiation was evaluated by measuring intracellular TGF-*β*1, *α*-smooth muscle actin (*α*-SMA), collagen type I protein expression, and metalloproteinase (MMP)-9 release.

As depicted in Figure 4, both conditions from HIV-infected Jurkat cells (“cm” and cell-to-cell contact) significantly increased *α*-SMA (protein expression and mRNA levels, 1.2-fold-change) (Figures 4(b) and 4(c)), up to 4.3-fold intracellular TGF-*β*1 production (Figure 4(d)), and a threefold increase in collagen deposition (Figure 4(e)–4(h)) in LX-2. Furthermore, under these conditions, LX-2 exhibited significant downregulation of MMP-9 (4.3-fold) in the culture supernatant (Figure 4(i)). Taken together, these results indicate that HIV-infected Jurkat cells, through soluble mediators and cell-to-cell contact, can induce IL-6 secretion and transdifferentiation of LX-2.

### 3.4. Soluble Factors from Short-Term HCV-Infected Hepatocytes Increase Oxidative Stress, Cell Death, and Collagen Deposition during LX-2-to-HIV Infected Jurkat Cells Contact

**Figure 5:**
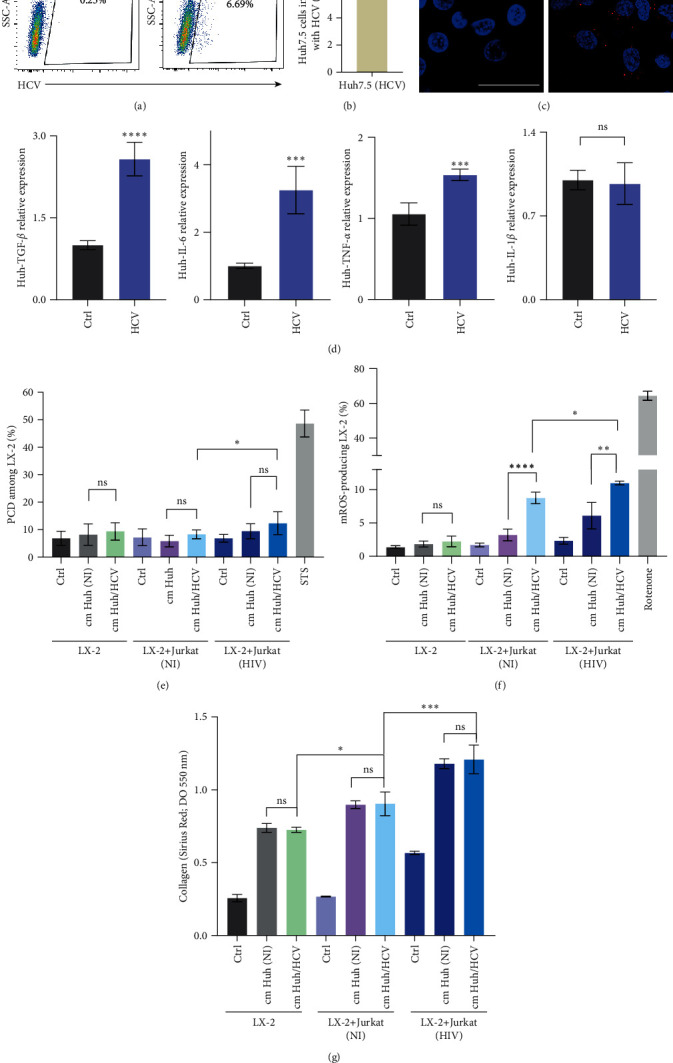
Determination of the role of HCV-infected hepatocytes on LX-2-to-HIV infected Jurkat cells contact. (a) Representative flow cytometry dot plots showing the relative percentages of Huh 7.5 infection after 72 hr. (b) Bar graph representing the percentage of infection obtained (7.2% ± 0.7%). (c) Determination of HCV infection of Huh7.5 cells by production by LX-2 revealed by immunofluorescence with a specific antibody. (d) Relative expression of TGF-*β*, IL-6, TNF-*α*, and IL-1*β* measured by RT-qPCR in Huh7.5 infected cells. (e) Programed cell death levels (PCD) (positive staining for annexin V and/or 7-AAD) among LX-2 cells, and LX-2-Jurkat coculture treated with “cm” from Huh7.5 cells during 72 hr. (f) mROS production by LX-2 cells and LX-2-Jurkat coculture treated with “cm” from Huh7.5 cells during 72 hr. (g) Determination of collagen deposition by LX-2, and LX-2–Jurkat coculture treated with “cm” from Huh7.5 cells during 72 hr, revealed by Sirius Red staining and quantified by OD readings at 550 nm. NS, not significant; STS, staurosporine. Graphics are showing values obtained from three independent experiments. Data are given as the mean ± SD,  ^*∗*^*p*  < 0.05,  ^*∗∗*^*p*  < 0.01,  ^*∗∗∗*^*p*  < 0.001, and  ^*∗∗∗∗*^*p*  < 0.0001.

Hepatitis C virus (HCV)-infected hepatocytes are known to release TGF-*β*1. Moreover, HCV proteins have been shown to directly trigger HSCs activation by modulating signaling and metabolic pathways [23]. To enhance the understanding of HCV/HIV coinfection-induced hepatic fibrosis, the LX-2/Jurkat coculture system was exposed to the “cm” from HCV-infected Huh 7.5 cells. For this goal, Huh7.5 cells were infected with HCV for 3 days (MOI = 1) reaching an efficiency of 7.2% ± 0.7% (Figure 5(a)–5(c)). At that time, “cm” from HCV-infected and noninfected Huh7.5 cells were collected, and total RNA was extracted. The latter was used to quantify mRNA levels of key cytokines involved in hepatic fibrosis. Thus, using RT-qPCR analysis, the HCV-infected Huh7.5 cells exhibited significantly higher mRNA levels of TGF-*β* (2.6-fold), TNF-*α* (1.5-fold), and IL-6 (3.2-fold), but not IL-1*β*, compared to uninfected control cells (Figure 5(d)). However, at 3 dpi, cytokine levels in the culture media were undetectable.

LX-2 cells were then exposed to HIV-infected Jurkat cells with or without “cm” from HCV-infected Huh7.5 cells, focusing on programed cell death (PCD), mROS production, and collagen deposition. The presence of “cm” from Huh7.5 cells, whether HCV-infected or not, did not significantly alter LX-2 cell viability under the three experimental conditions. However, PCD levels in LX-2 cells moderately increased when in contact with HIV-infected Jurkat cells and soluble factors (including HCV) from HCV-infected Huh7.5 cells (Figure 5(e)).

mROS levels in LX-2 cells significantly increased when exposed to a conditioned medium from HCV-infected Huh7.5 cells, but only in the presence of cell-to-cell contact with Jurkat cells, infected with HIV or not (1.8-fold and 2.7-fold, respectively). Simultaneous exposure of LX-2 cells to a conditioned medium from HCV-infected Huh7.5 cells and cell-to-cell contact with HIV-infected Jurkat cells significantly increased mROS production (1.25-fold) (Figure 5(f)).

Examining collagen deposition by LX-2 cells revealed that the sole presence of soluble mediators from HCV-infected Huh7.5 cells did not lead to extracellular matrix production. However, in the presence of soluble factors from HCV-infected Huh7.5 cells, collagen deposition significantly increased when LX-2 cells were in contact with Jurkat cells, further pronounced when the lymphoid cells were HIV-infected (Figure 5(g)).

In summary, ROS levels among LX-2 cells is increased when simultaneously in contact with HIV-infected lymphocytes, and their surrounding microenvironment includes soluble factors from HCV-infected hepatocytes. Mere exposure to soluble mediators from short-term HCV-infected hepatocytes does not appear to affect LX-2 cells' profibrotic potential.

## 4. Discussion

Individuals coinfected with HIV and HCV, exhibiting ongoing HIV viremia, demonstrate accelerated rates of fibrosis progression related to HCV. Successful suppression of HIV with antiretroviral therapy leads to a reduction in fibrosis progression rates and necroinflammatory activity. This observation implies an accelerating influence of HIV on the progression of fibrosis [24].

In this study, in vitro coculture and conditioned medium encompassing three cell types crucial in the development of hepatic fibrosis, HSCs, represented by LX-2, and two cell lines that support active replication of HIV and HCV, namely, CD4 T lymphocytes (Jurkat cells) and hepatocytes (Huh7.5), were used. This system provides insights into potential cellular and molecular mechanisms through which HIV, by influencing activated HSCs, may exacerbate liver injury in chronic liver diseases. However, these findings contradict the assumption that HSCs are susceptible to HIV infection [25]. Utilizing the detection of intracellular HIV-capsid antigen by flow cytometry, as well as its release into the extracellular medium through enzyme-linked immunosorbent assay (ELISA), no infection in HSCs, either by encountering free virus or by engaging in intercellular contact with infected CD4 T lymphocytes, was observed.

In this context, it was observed that the expression of the CD4 receptor in LX-2 cells when coculture with HIV-infected cells may be associated with the reported HSCs activation, fibrogenesis, and proliferation [26]. As recently reported by the authors, when the infection was conducted using pseudotyped HIV incorporating the glycoprotein G from vesicular stomatitis virus (VSV-G) in a CD4/CXCR4/CCR5-independent manner, it was demonstrated that LX-2 cells exhibit permissiveness to intracellular HIV replication [27, 28].

Taking into account the lack of HIV infection in HSCs, the likelihood of bystander effects from HIV-infected lymphoid cells is conceivable given that a higher intracellular ROS level, and cytokines (IL-2 and TGF-*β*) were measured. These effects are presumed to be facilitated through both direct cell-to-cell contact and the release of soluble factors. Initially, mainly through cell-to-cell contact and not the cell-free virus, HIV-infected lymphocytes induced redox imbalance in HSCs, resulting in increased ROS production that paradoxically did not induce higher levels of cellular death. This phenomenon occurred soon after contact and also after 3 days of interaction. These results align with previous studies showing elevated levels of reactive oxygen species (ROS) prompting the transdifferentiation of quiescent HSCs into an activated, highly proliferative myofibroblast-like phenotype that has increased ROS-detoxifying capacity compared to quiescent HSCs [21]. Additionally, ROS stimulates the synthesis of extracellular matrix (ECM) by activated HSCs [20, 29] with such interactions between ROS and HSCs emerging as contributors to excessive deposition of ECM. Furthermore, this oxidative stress directly contributes to inflammatory responses by inducing nuclear factor-*κ*B, thereby elevating the levels of IL-6 and TGF-*β* [30].

In the experimental conditions tested, the high efficiency of HIV infection in lymphocytes led to an increased rate of apoptosis. Originating from hepatocytes, these apoptotic bodies have been demonstrated to constitute a profibrotic stimulus after being engulfed by HSCs [31, 32]. Subsequently, HIV-infected lymphocytes/LX-2 communication, both through intercellular contact and soluble mediators, resulted in a significant increase in the levels of both released IL-6 and intracellular TGF-*β* in LX-2 cells. These cytokines play a pivotal role not only in the development of fibrosis but also in hepatocyte regeneration and endogenous inflammasome activation [33, 34]. Afterward, the LX-2-to-HIV infected Jurkat microenvironment gave rise to alterations in extracellular matrix expression and turnover, such as an augmentation in collagen deposition and the expression of *α*-SMA, alongside a decrease in MMP-9, a protein involved in matrix remodeling.

Finally, the role of the hepatitis C virus (HCV)/HIV copresence in LX-2 alteration and its contribution to hepatic fibrosis was evaluated. HCV-infected hepatocytes (Huh7.5) exhibited significantly increased levels of profibrotic gene transcriptions TGF-*β*, TNF-*α*, and IL-6, in accordance with previous observations [35–38]. However, by employing the conditioned medium obtained from short-term HCV-infected hepatocytes, the soluble factors released from these hepatocytes did not alter cell death, oxidative stress, or collagen production in LX-2 cells. Nevertheless, the HCV incumbency on ROS imbalance was significantly higher when LX-2 cells were in contact with HIV-infected Jurkat cells.

This study has some limitations. Firstly, the experiments primarily utilized cell lines instead of primary human cells. They may present unstable cell properties within a cell population and different phenotypes from liver cells in vivo and alter physiological properties. To minimize these effects, a short number of passaging was used. Secondly, the X4-NL4-3 HIV and J6/JFH HCV strains adapted for laboratory conditions exhibit lower predictability for in vivo outcomes compared to strains facilitating transmission among infected individuals. Nevertheless, these findings necessitate further validation in in vivo models. Thirdly, the observed phenotypic effects lack complete support from gene expression profiles, partly due to the chosen time point, inherent cell population heterogeneity, and concurrent expression of key markers. Future studies will focus on investigating these markers and their interactions at the protein level.

This study provides insight into the mechanistic aspects underlying increased fibrogenesis in individuals coinfected with HCV/HIV compared to those solely infected with HCV.

## Data Availability

The raw data supporting the conclusions of this article will be made available by the authors, without undue reservation.
